# Association between chronic kidney disease stages and changes in ambulatory blood pressure monitoring parameters

**DOI:** 10.1590/2175-8239-JBN-2023-0066en

**Published:** 2024-06-17

**Authors:** André Murad Nagahama, Vanessa dos Santos Silva, Vanessa Burgugi Banin, Roberto Jorge da Silva Franco, Pasqual Barretti, Silmeia Garcia Zanati Bazan, Luis Cuadrado Martin

**Affiliations:** 1 Universidade Estadual Paulista “Júlio de Mesquita Filho”, Faculdade de Medicina, Botucatu, SP, Brazil.

**Keywords:** Chronic Kidney Disease, Chronic Kidney Failure, Ambulatory Blood Pressure Monitoring, Hypertension, Arterial Hypertension, Circadian Rhythm

## Abstract

**Introduction:**

Blood pressure (BP) assessment affects the management of arterial hypertension (AH) in chronic kidney disease (CKD). CKD patients have specific patterns of BP behavior during ambulatory blood pressure monitoring (ABPM).

**Objectives:**

The aim of the current study was to evaluate the associations between progressive stages of CKD and changes in ABPM.

**Methodology:**

This is a cross-sectional study with 851 patients treated in outpatient clinics of a university hospital who underwent ABPM examination from January 2004 to February 2012 in order to assess the presence and control of AH. The outcomes considered were the ABPM parameters. The variable of interest was CKD staging. Confounding factors included age, sex, body mass index, smoking, cause of CKD, and use of antihypertensive drugs.

**Results:**

Systolic BP (SBP) was associated with CKD stages 3b and 5, irrespective of confounding variables. Pulse pressure was only associated with stage 5. The SBP coefficient of variation was progressively associated with stages 3a, 4 and 5, while the diastolic blood pressure (DBP) coefficient of variation showed no association. SBP reduction was associated with stages 2, 4 and 5, and the decline in DBP with stages 4 and 5. Other ABPM parameters showed no association with CKD stages after adjustments.

**Conclusion:**

Advanced stages of CKD were associated with lower nocturnal dipping and greater variability in blood pressure.

## Introduction

The prevalence of arterial hypertension (AH) is high^1^. This clinical condition compromises the heart, blood vessels, brain, and kidneys^2^. Chronic kidney disease (CKD) prevalence in Brazil is estimated at around 10% of the population^2^, of which approximately 150,000 are in the most advanced progressive stage, requiring dialysis^3^.

AH is highly prevalent in CKD^4^. The pathophysiological link between AH and CKD is well known, with AH being the major risk factor for CKD in Brazil^3^. Hypertension and kidney injury establish a cycle of cause and consequence that worsens the prognosis^5^. Among the pathophysiological characteristics of AH in CKD, we could mention sodium retention, sympathetic hyperactivity, and hyperactivity of the renin-angiotensin-aldosterone system^6^. These particularities are associated with characteristics of blood pressure (BP) behavior and its circadian pattern^7^.

Thus, BP control is crucial as it reduces structural and functional damage to nephrons and improves the life expectancy of patients with kidney disease^6^. This emphasizes the importance of accurate BP assessment in CKD. Defining the type of AH as normotension, true hypertension, white-coat hypertension, and masked hypertension is paramount for patient management^8^.

Ambulatory blood pressure monitoring (ABPM) is the best available means of assessing patients’ BP over a 24-hour period in the clinic^8,9,10,11,12^. CKD patients have specific patterns of BP behavior over a 24-hour period, including reduced nocturnal dipping or even increased BP during sleep.

Some studies have evaluated these patterns; however, they are still scarce. A meta-analysis^13^ identified only 6 studies^14,15,16,17,18,19^ that assessed the prevalence of white-coat hypertension and masked hypertension in chronic kidney disease patients. This meta-analysis identified a high prevalence of masked hypertension and white-coat hypertension in CKD patients. However, among the analyzed studies, the most significant one had included only 290 patients^15^. It is important to note that these studies did not consider confounding variables.

We did not identify any studies on ABPM specific alterations in CKD that separately evaluated each progressive stage of the disease, compared them with control groups without CKD, or performed statistical adjustments for confounding variables.

Therefore, the aim of the current study was to assess the associations between progressive stages of CKD and changes in ABPM, considering confounding variables.

## Methods

In this cross-sectional study, we analyzed 851 patients from the cardiology, endocrinology, internal medicine and nephrology outpatient clinics at *Hospital das Clínicas*, Botucatu Medical School. These patients underwent ABPM testing from January 2004 to February 2012, which is recommended for diagnostic assessment and control of AH^20^. The majority of patients were on drug treatment, and this was considered in the statistical analyses.

The outcomes considered were the following ABPM parameters: the average of systolic blood pressure (SBP), diastolic blood pressure (DBP), and pulse pressure (PP); nighttime SBP and DBP dipping percentage; BP variability; AH phenotype (normotension, sustained hypertension, white-coat hypertension and masked hypertension). The variable of interest is CKD staging. We considered confounding factors: age, sex, body mass index (BMI), smoking, and cause of CKD.

Inclusion criteria comprised patients aged 18 years and older who underwent ABPM during the study period. The exclusion criteria were: patients who underwent technically inadequate ABPMs according to the V Brazilian Guidelines for Ambulatory Blood Pressure Monitoring and Home Blood Pressure Monitoring Guidelines; pregnant women; kidney transplant recipients; patients with Parkinson’s disease; patients with atrial fibrillation; incomplete ABPM data; repeated tests; and lack of data to establish the CKD stage.

Office SBP and DBP obtained immediately before the ABPM examination were recorded. Creatinine levels and urine tests conducted within a maximum of 3 months before or after the ABPM were recorded. Glomerular filtration rate (GFR) was calculated using the Chronic Kidney Disease Epidemiology Collaboration (CKD-EPI) formula.

ABPM tests were performed using the Spacelabs^®^ 90202. ABPM was standardized based on the V Brazilian Guidelines for Ambulatory Blood Pressure Monitoring (ABPM) and the III Guidelines for Home Blood Pressure Monitoring^21^. The average SBP and DBP were recorded via software over the entire examination, during wakefulness and sleep period. SBP and DBP dipping during the sleep period were calculated. Blood pressure variability was quantified by the standard deviation of pressures, as well as by the coefficient of variation.

This study complies with the Strobe Statement^22^, and with Resolution 466/12 of the National Health Council, which is compatible with the Declaration of Helsinki. The local research ethics committee also approved it. Informed consent was waived.

## Definitions

We used the following as a definition for white-coat hypertension: office blood pressure greater than or equal to 140/90 mmHg and ABPM measurements during daytime period less than or equal to 135/85 mmHg. In light of the new considerations of the VI Brazilian Guidelines for Ambulatory Blood Pressure Monitoring (ABPM)^23^, nighttime BP was also considered. Thus, the definition included a 24-hour BP average greater than or equal to 130/80 mmHg.

The definition of masked hypertension used was: office BP less than 140/90 mmHg and ABPM measurements during awake hours greater than or equal to 135/85 mmHg. Given the new considerations of the VI Brazilian Guidelines for Ambulatory Blood Pressure Monitoring^23^, BP during sleep hours was also considered. Therefore, the definition included BP in the sleep period greater than or equal to 120/70 mmHg.

Sustained hypertension was defined when office BP was greater than or equal to 140/90 mmHg and daytime ABPM measurements were greater than or equal to 135/85 mmHg; whereas normotension was defined when office BP was less than 140/90 mmHg and daytime ABPM measurements were less than 135/85 mmHg. The data were also reanalyzed following the VI Brazilian Guidelines for Ambulatory Blood Pressure Monitoring^23^.

CKD was defined by KDIGO 2012^24^ as a glomerular filtration rate lower than 60 mL/min/1.73m^2^, or the presence of proteinuria for more than 3 months. Additionally, CKD was classified based on its etiology and the GFR.

The different stages of GFR in CKD are: G1 defined by a GFR ≥ 90 mL/min/1.73m^2^. G2, GFR between 60 and 89 mL/min/1.73m^2^. G3a, GFR between 45 and 59 mL/min/1.73m^2^. G3b is characterized by a GFR between 30 and 44 mL/min/1.73m^2^. G4, GFR between 15 and 29 mL/min/1.73m^2^. Finally, G5 is defined by a GFR lower than 15 mL/min/1.73m^2^.

## Statistical Analysis

Categorical data were expressed as absolute numbers and frequencies. Non-categorical variables with normal distribution were expressed as mean ± standard deviation. Data normality was confirmed using the Kolmogorov-Smirnov test. Continuous variables with normal distribution were compared using one-way ANOVA. Continuous variables with non-Gaussian distributions were subjected to the Kruskal-Wallis test. Categorical variables were compared using the χ^2^ test. The multiple generalized linear regression model was applied to the variable of interest, as well as to the following confounding factors: age, sex, BMI, smoking, cause of CKD and number of antihypertensive drug classes. Data were discussed considering the significance level of p < 0.05.

## Results

**Figure 1.**
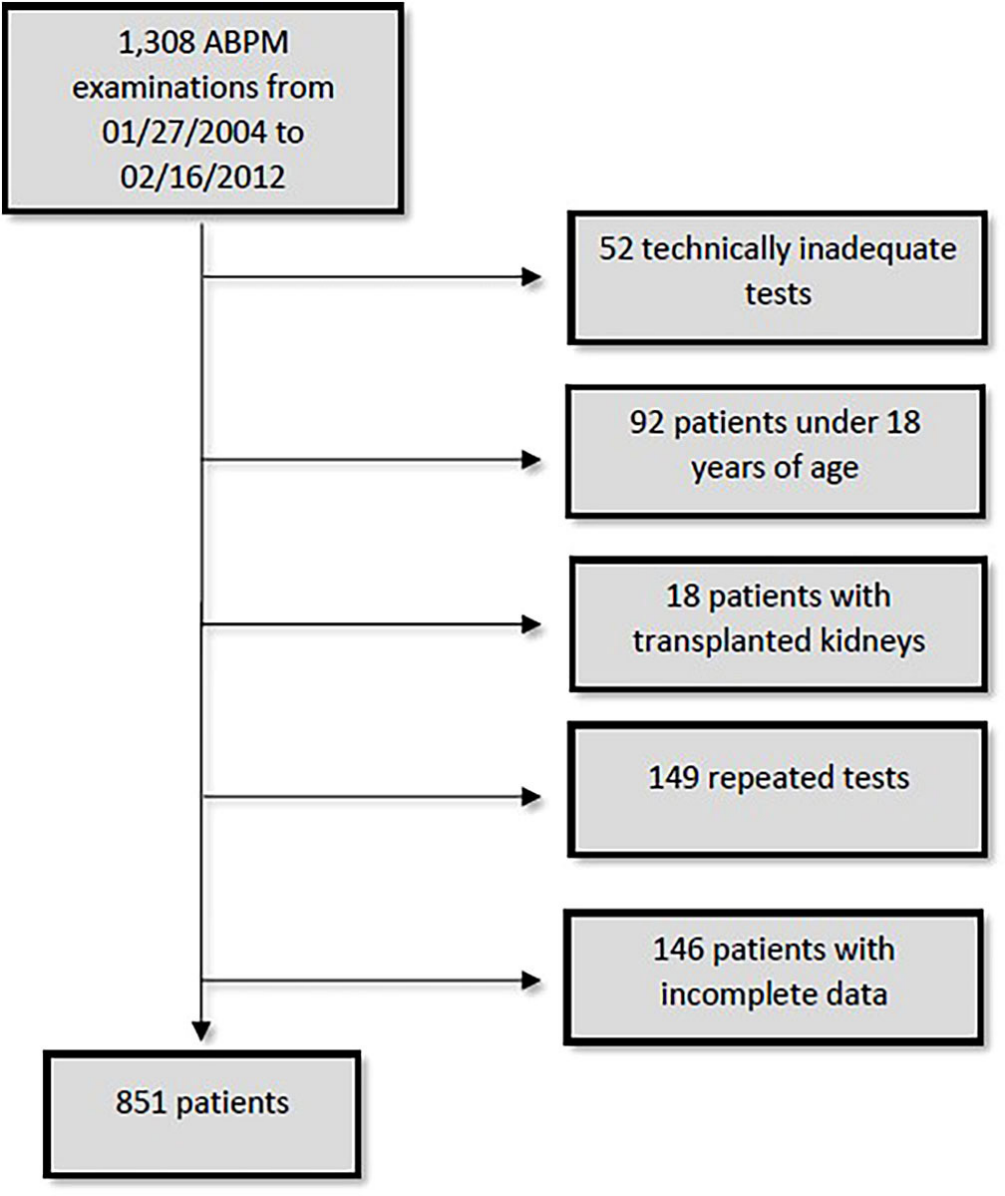
Patient inclusion flowchart.

A total of 1,308 ABPM exams were performed over the study period. Of these, 52 were excluded as they failed to meet the technical conditions for validating the procedure. In addition, 92 patients were under 18 years of age, 18 were kidney transplant patients and 149 were repeated examinations, which were also excluded. Finally, an additional 146 patients were excluded due to incomplete creatinine data or urine test results. Thus, 851 patients were included in the analyses (Figure 1).

Age was 55 ± 16.2 years; 42.4% were male; 6.6% were Afro-descendants; Asians and Caucasians accounted for 0.9% and 92.5%, respectively. Out of 682 patients, 74 (10.9%) were active smokers, while 156 (22.9%) were former smokers.

From the total sample, 475 patients had CKD. Among these 475 CKD patients, 168 (35.6%) had diabetes as the cause, 227 (47.8%) had hypertension, 32 (6.7%) had glomerular causes, and 47 (9.9%) had other causes.

As for CKD staging, 376 out of 851 patients did not have CKD. Among the 475 CKD patients, 45 were in stage G1; 54 in stage G2; 97 in stage G3a; 89 in stage G3b; 71 in stage G4; and 119 were in stage G5 (Table 1).

**Table 1.** Clinical and demographic data of 851 patients undergoing ambulatory blood pressure monitoring

	No CKD (n = 376)	CKD stages						P
		1 (n = 45)	2 (n = 54)	3a (n = 97)	3b (n = 89)	4 (n = 71)	5 (n = 119)	
Age (years)	49 ± 15.0	40 ± 13.8	54 ± 13.7	61 ± 13.6	66 ± 14.7	65 ± 11.6	61 ± 14.2	<0.001
Male (%)	40	24	38	45	49	49	54	0.010
Afro-descendants (%)	7	11	No CKD	4	4	7	10	0.154
BMI (Kg/m^2^)	29 ± 5.3	27 ± 5.9	29 ± 6.8	28 ± 6.0	28 ± 6.6	28 ± 6.9	25 ± 3.7	0.344
Active smokers *(%)	34/293 (11)	5/33 (15)	1/42 (2)	3/74 (4)	6/73 (8)	11/60 (18)	14/107 (13)	0.062
Former smokers *(%)	52/293 (17)	7/33 (21)	12/42 (28)	23/74 (31)	24/73 (32)	13/60 (21)	24/107 (22)	0.056
Diabetics (%)	21	20	46	38	31	42	49	<0.001
Dyslipidemia *(%)	102/281 (36)	11/31(35)	16/45 (35)	46/78 (58)	49/77 (63)	43/63 (68)	45/91 (49)	<0.001

Abbreviations – CKD: chronic kidney disease; BMI: body mass index;

* (%) = positive cases/total cases with available data (%).

**Figure 2.**
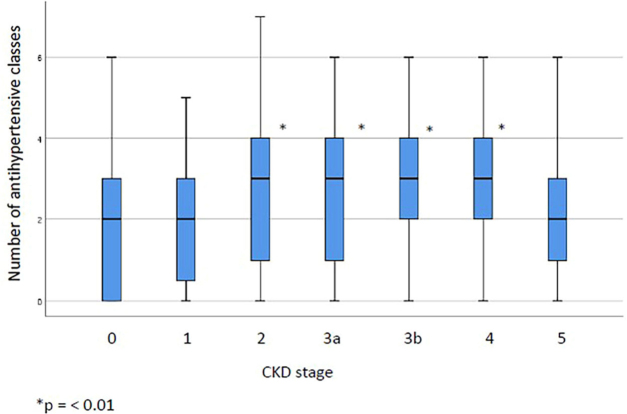
Box plot of the distribution of antihypertensive drugs across different stages of chronic kidney disease.

Chronic kidney disease staging was associated with age (p < 0.001), male sex (p = 0.01), diabetes (p < 0.001) and dyslipidemia (p < 0.001). Ethnicity, BMI (p = 0.344), and smoking (p = 0.059) had no statistically significant association with CKD progression (Table 1), but were included in the multiple analysis model. The median and interquartile range of the number of antihypertensive classes used at each stage are depicted in Figure 2.

By means of ABPM, it was possible to observe the following distribution of hypertension phenotypes: 230 were normotensive; 140 had masked hypertension; 134 had white-coat hypertension; 347 were true hypertensives (Table 2).

**Table 2.** Blood pressure results of 851 patients undergoing ambulatory blood pressure monitoring

	No CKD (n = 376)	CKD stages						P
		1 (n = 45)	2 (n = 54)	3a (n = 97)	3b (n = 89)	4 (n = 71)	5 (n = 119)	
Office SBP (mmHg)	137 ± 22.1	137 ± 24.8	137 ± 21.9	141 ± 24.5	141 ± 25.6	143 ± 27.4	148 ± 28.9	0.007
Office DBP (mmHg)	89 ± 14.3	87 ± 13.3	86 ± 13.4	87 ± 16.4	84 ± 17.7	84 ± 15.9	87 ± 16.1	0.156
24h SBP (mmHg)	129 ± 14.6	127 ± 15.7	131 ± 18.0	129 ± 15.9	128 ± 16.6	134 ± 22.3	141 ± 23.3	<0.001
24h SBP CV (%)	10.1 ± 2.40	9.7 ± 2.38	10.3 ± 2.74	11.1 ± 3.03	10.9 ± 3.19	11.8 ± 3.60	11.9 ± 3.24	<0.001
24h DBP (mmHg)	79 ± 11.1	77 ± 10.3	78 ± 10.6	76 ± 9.9	73 ± 13.0	76 ± 13.0	79 ± 14.7	0.001
24h DBP CV (%)	13.9 ± 3.21	14.5 ± 3.38	13.6 ± 3.12	14.3 ± 3.57	14.0 ± 3.26	13.9 ± 3.53	13.7 ± 3.74	0.723
24h PP (mmHg)	50 ± 10.3	49 ± 11.1	52 ± 13.8	52 ± 12.1	54 ± 13.2	58 ± 15.8	61 ± 16.6	<0.001
Daytime SBP (mmHg)	132 ± 14.9	130 ± 15.8	133 ± 17.6	131 ± 16.0	130 ± 16.6	135 ± 21.7	141 ± 23.0	<0.001
Daytime DBP (mmHg)	82 ± 11.5	81 ± 11.0	81 ± 11.4	79 ± 10.6	76 ± 13.4	78 ± 13.2	80 ± 14.8	0.001
Nighttime SBP (mmHg)	121 ± 15.8	119 ± 16.5	126 ± 20.4	123 ± 18.7	122 ± 18.0	131 ± 27.0	139 ± 26.1	<0.001
Nighttime DBP (mmHg)	71 ± 11.8	69 ± 10.0	73 ± 11.0	70 ± 11.1	68 ± 13.7	71 ± 14.9	77 ± 16.1	<0.001
SBP dip (%)	8 ± 7.1	8 ± 7.5	5 ± 7.6	5 ± 10.1	5 ± 6.0	3 ± 10.8	1 ± 7.5	<0.001
DBP dip (%)	13 ± 8.2	15 ± 7.6	9 ± 9.9	10 ± 9.9	10 ± 7.7	7 ± 12.1	4 ± 7.8	<0.001
SBP SD (mmHg)	13 ± 3.4	12 ± 3.3	13 ± 4.6	14 ± 3.9	13 ± 4.4	15 ± 5.3	16 ± 4.7	<0.001
DBP SD (mmHg)	10 ± 2.3	11 ± 2.6	10 ± 2.3	10 ± 2.6	10 ± 2.5	10 ± 2.7	10 ± 2.3	0.239
Phenotypes								
Normotensive (%)	81(21)	9(20)	10(19)	21(22)	23(26)	15(21)	13(11)	0.186
MH (%)	93(25)	13(29)	16(30)	14(14)	17(19)	20(28)	25(21)	0.180
WCH (%)	41(11)	6(13)	4(7)	24(25)	12(13)	2(3)	7(6)	<0.001
Hypertensive (%)	161(43)	17(38)	24(44)	38(39)	37(42)	34(48)	74(62)	0.006
Office blood pressure accuracy (%)	64	58	63	61	67	69	73	0.415

Abbreviations – CKD: chronic kidney disease; SBP: systolic blood pressure; DBP: diastolic blood pressure; CV: coefficient of variation; PP: pulse pressure; SD: standard deviation; MH: masked hypertension; WCH: white-coat hypertension. Traditional phenotype: 5th ABPM Guideline; current phenotype: 6th ABPM Guideline.

Regarding BP differences across the progressive stages of CKD (Table 2), there was a progressive elevation in office SBP with the progression of the kidney disease (p = 0.007). However, office DBP was not associated with CKD progression (p = 0.156). Except for the coefficient of variation of 24-hour DBP and DBP standard deviation, we observed an association between different stages of CKD progression and worsening of ABPM test parameters. There was an association between hypertension phenotypes and the stage of CKD for white-coat hypertension and sustained hypertension. The accuracy of office blood pressure was not associated with more advanced stages of CKD (Table 2).

By performing generalized linear regressions, we obtained a number of associations between CKD stage and ABPM test results adjusted for confounding variables: sex, smoking, dyslipidemia, diabetes, age, and number of antihypertensive drug classes. Systolic blood pressure on ABPM was associated with CKD stages 3a and 5, regardless of the confounding variables included in the model (Table 3). There was a decrease in the 24-hour SBP value for stage 3b, while an increase in its value was observed for stage 5. The decrease in diastolic blood pressure on ABPM was associated only with stage 3b. The increase in 24-hour PP on ABPM showed a statistically significant association with stage 5 CKD, irrespective of the adjustment variables (Table 3).

**Table 3.** General linear regression model with systolic and diastolic blood pressure, and 24-hour pulse pressure of 851 patients undergoing ambulatory blood pressure monitoring as the outcome variable, adjusted for age, sex, smoking, diabetes, dyslipidemia, and number of antihypertensive classes

	β	95% Wald Confidence Interval		p
		Inferior	Superior	
SBP				
Stage 5	5.421	0.958	9.885	0.017
Stage 4	–0.362	–5.646	4.921	0.893
Stage 3b	–6.043	–11.028	–1.058	0.018
Stage 3a	–2.829	–7.605	1.947	0.246
Stage 2	–0.690	–6.667	5.288	0.821
Stage 1	0.332	–7.568	6.923	0.929
No CKD	Reference			
DBP				
Stage 5	0.578	–2.449	3.605	0.708
Stage 4	–2.469	–6.042	1.124	0.179
Stage 3b	–3.751	–7.132	–0.371	0.030
Stage 3a	–1.135	–4.373	2.104	0.492
Stage 2	–0.625	–4.679	3.428	0.762
Stage 1	–3.365	–8.285	1.554	0.180
No CKD	Reference			
PP				
Stage 5	4.843	1.802	7.884	0.002
Stage 4	2.096	–1.506	5.696	0.254
Stage 3b	–2.292	–5.688	1.104	0.186
Stage 3a	–1.694	–4.948	1.559	0.307
Stage 2	–0.064	–4.136	4.008	0.975
Stage 1	3.034	–1.908	7.975	0.229
Stage 0	Reference			

An increase in the coefficient of variation of 24-hour SBP was associated with stages 3a, 4 and 5 of CKD in the regressions, even considering the other confounding variables. Nevertheless, the coefficient of variation of 24-hour DBP on ABPM was not associated with any stage of CKD (Table 4).

**Table 4.** General linear regression model with systolic and diastolic blood pressure coefficient of variation and systolic and diastolic blood pressure dipping of 851 patients undergoing ambulatory blood pressure monitoring as the outcome variable, adjusted for age, sex, smoking, diabetes and dyslipidemia, and number of antihypertensive classes

	β	95% Wald Confidence Interval		p
		Inferior	Superior	
SBP CV				
Stage 5	2.032	1.282	2.782	<0.001
Stage 4	1.138	0.250	2.026	0.001
Stage 3b	0.155	–0.683	0.993	0.716
Stage 3a	0.977	0.175	1.780	0.017
Stage 2	0.213	–0.791	1.218	0.677
Stage 1	–0.171	–1.390	1.049	0.784
No CKD	Reference			
DBP CV				
Stage 5	0.778	–0.137	1.693	0.095
Stage 4	0.484	–0.598	1.567	0.380
Stage 3b	0.437	–0.584	1.459	0.401
Stage 3a	0.655	–0.324	1.634	0.190
Stage 2	–0.232	–1.457	0.993	0.711
Stage 1	0.138	–1.348	1.625	0.855
No CKD	Reference			
SBP Dipping				
Stage 5	–4.272	–6.306	–2.238	<0.001
Stage 4	–4.494	–6.901	–2.087	<0.001
Stage 3b	–1.323	–3.595	0.948	0.253
Stage 3a	0.253	–1.923	2.429	0.820
Stage 2	–2.997	–5.720	–0.274	0.031
Stage 1	–1.152	–4.457	2.153	0.494
No CKD	Reference			
DBP Dipping				
Stage 5	–5.370	–7.694	–3.046	<0.001
Stage 4	–3.576	–6.327	–0.825	0.011
Stage 3b	0.030	–2.566	2.629	0.982
Stage 3a	5.474	–1.940	3.034	0.667
Stage 2	–2.436	–5.548	0.677	0.125
Stage 1	–0.386	–4.164	3.391	0.841
No CKD	Reference			

Finally, regarding BP dipping over the course of examination: decreased SBP dipping were independently associated with stages 2, 4 and 5. However, a reduced value for DBP dipping was only associated with the two most advanced stages of CKD (Table 4).

## Discussion

This study examined the association between CKD stages and abnormalities observed in ABPM. It was possible to note that more advanced stages of CKD were progressively associated with lower nocturnal dipping and greater variability in blood pressure. 24-hour SBP and 24-hour PP were associated with a more advanced level of CKD (stage V). The distinctive feature of the current study is that all these associations were seen regardless of sex, age, smoking, diabetes, and even the number of antihypertensive drug classes.

A cross-sectional study with 10,271 hypertensive patients revealed that the pulse pressure of those with chronic kidney disease was significantly higher compared to the PP of hypertensive individuals without kidney disease^7^. In this study, the most significant difference between the two groups investigated was the prevalence of the “riser” pattern (17.6% in CKD patients *vs.* 7.1% in non-CKD patients), i.e. where the average of nighttime systolic blood pressure is higher than the average of systolic blood pressure during awake hours. These data are consistent with those of the current study; however, this cross-sectional study did not adjust for confounding variables such as sex, age, smoking, and the presence of diabetes.

Agarwal & Andersen^25^, in a study involving 232 veterans, considering 17 confounding variables, observed that SBP was not associated with a decline in glomerular filtration when the appropriate adjustments were made. However, the authors observed a strong association between 24-hour ABPM SBP and proteinuria. In addition, the dipper pattern generally decreased as the stage of CKD progressed, albeit not in a linear manner. In our study, the non-dipper pattern was associated with a decline in glomerular filtration rate, i.e. with more advanced stages of CKD (stages 2, 4 and 5), consistent with expectations based on literature evidence^26,27^. In our study, patients in stages 3b and 5 showed an association with lower and higher SBP, respectively. It is interesting to note that the study by Agarwal & Andersen did not include dialysis patients (stage 5)^25^.

BP variability in chronic kidney patients is increased, which is believed to occur due to a malfunction of baroreceptors and uncontrolled balance between sympathetic and parasympathetic activity^28^. Timio et al.^29^, in a prospective study from 1993, observed that blood pressure variability in renal patients was higher than in controls. In our study, when performing generalized linear regression, the SBP coefficient of variation was only associated with the more advanced stages of CKD (stages 3a, 4 and 5), whereas for DBP, no association was observed at any stage of the studied disease.

Paradoxically, in the present study, the accuracy of office blood pressure measurements was not linked to more severe CKD staging. This finding holds even when considering both old and new classifications for masked hypertension and white-coat hypertension, which address the use of nighttime and daytime ABPM blood pressures differently. We believe that this discrepancy with the literature^30^ is due to a standardized BP measurement performed in our study, reducing possible external interferences such as caffeine consumption, smoking, physical exercise, and negligence regarding resting time before measurement.

We should acknowledge some limitations of this work. The subjects in this study were all from the region of Botucatu, a mid-sized city within the state of São Paulo. Consequently, the demographic sample obtained does not reflect the demographic parameters of Brazil as a whole. We believe this is due to the characteristics of the specific city involved. We also had no means of assessing adherence to AH and CKD treatment, although we were aware of each patient’s therapeutic regimen. Another limiting factor in this study was that, although we had the qualitative urine test assessed by Urinalysis, we did not collect data on quantitative proteinuria from a substantial number of patients, which prevented a complete KDIGO 2012 classification. Finally, the prevalence of masked hypertension may have been underestimated, considering that in this study, ABPM was not systematically performed on all chronic kidney disease patients with normal office blood pressure, but only on those who underwent ABPM at our institution. Conversely, the same bias could occur among patients who did not present with CKD in our sample, which could balance the biases.

We can also highlight positive aspects of the current study that have not yet been explored in literature. Firstly, since this was a cross-sectional study, we were able to encompass a large sample of 851 patients with good representativeness across the different stages of CKD. A review of the literature revealed a paucity of studies that have conducted such a comprehensive analysis of ABPM parameters in conjunction with different stages of CKD. Furthermore, few studies have considered confounding variables when analyzing their data.

## Conclusion

In our sample, more advanced stages of CKD were associated with reduced nocturnal dipping and greater blood pressure variability. Elevations in SBP and DBP were only observed in stage 5. Conversely, the decrease in 24-hour SBP was associated with an intermediate CKD stage (stage 3b), even considering confounding factors. Paradoxically, the accuracy of office-measured blood pressure was not associated with more advanced stages of CKD.

In this context, the current study encompassed a larger number of patients and data for analysis, as well as a detailed assessment of the relationships between different stages of CKD and the main specific characteristics of ABPM, while considering potential confounding factors.

This allowed us to properly explore how the different stages of CKD are associated with the parameters of ABPM examination.

## References

[1] Barroso WKS, Rodrigues CIS, Bortolotto LA, Mota-Gomes MA, Brandão AA, Feitosa ADM (2021). Diretrizes Brasileiras de Hipertensão Arterial –
2020.. Arq Bras Cardiol..

[2] Piccolli AP, Nascimento MMD, Riella MC (2017). Prevalence of chronic kidney disease in a population in southern
Brazil (Pro-Renal Study).. J Bras Nefrol.

[3] Nerbass FB, Lima HDN, Thomé FS, Vieira No OM, Sesso R, Lugon JR (2023). Brazilian dialysis survey 2021.. J Bras Nefrol.

[4] Muntner P, Anderson A, Charleston J, Chen Z, Ford V, Makos G (2010). Chronic Renal Insufficiency Cohort (CRIC) Study Investigators.
Hypertension awareness, treatment, and control in adults with CKD: results
from the Chronic Renal Insufficiency Cohort (CRIC) study.. Am J Kidney Dis.

[5] Cameron JS. (1999). Villain and victim: the kidney and high blood pressure in the
nineteenth century.. J R Coll Physicians Lond.

[6] Kidney Disease: Improving Global Outcomes (KDIGO) Blood Pressure
Work Group. (2021). KDIGO 2021 clinical practice guideline for the management of
blood pressure in chronic kidney disease.. Kidney Int..

[7] Mojón A, Ayala DE, Piñeiro L, Otero A, Crespo JJ, Moyá A (2013). Hygia Project Investigators. Comparison of ambulatory blood
pressure parameters of hypertensive patients with and without chronic kidney
disease.. Chronobiol Int..

[8] Velasquez MT, Beddhu S, Nobakht E, Rahman M, Raj DS (2016). Ambulatory blood pressure in chronic kidney disease: ready for
prime time?. Kidney Int Rep.

[9] Agarwal R, Andersen MJ (2006). Prognostic importance of ambulatory blood pressure recordings in
patients with chronic kidney disease.. Kidney Int.

[10] Dolan E, Stanton A, Thijs L, Hinedi K, Atkins N, McClory S (2005). Superiority of ambulatory over clinic blood pressure measurement
in predicting mortality: the Dublin outcome study.. Hypertension.

[11] Wen RW, Chen XQ, Zhu Y, Ke JT, Du Y, Wang C (2020). Ambulatory blood pressure is better associated with target organ
damage than clinic blood pressure in patients with primary glomerular
disease.. BMC Nephrol..

[12] Aslam N, Missick S, Haley W (2019). Ambulatory blood pressure monitoring: profiles in chronic kidney
disease patients and utility in management.. Adv Chronic Kidney Dis.

[13] Bangash F, Agarwal R (2009). Masked hypertension and white-coat hypertension in chronic kidney
disease: a meta-analysis.. Clin J Am Soc Nephrol.

[14] Andersen MJ, Khawandi W, Agarwal R (2005). Home blood pressure monitoring in CKD.. Am J Kidney Dis.

[15] Minutolo R, Borrelli S, Scigliano R, Bellizzi V, Chiodini P, Cianciaruso B (2007). Prevalence and clinical correlates of white coat hypertension in
chronic kidney disease.. Nephrol Dial Transplant.

[16] Matsui Y, Eguchi K, Ishikawa J, Hoshide S, Shimada K, Kario K (2007). Subclinical arterial damage in untreated masked hypertensive
subjects detected by home blood pressure measurement.. Am J Hypertens.

[17] Kuriyama S, Otsuka Y, Iida R, Matsumoto K, Hosoya T (2007). Morning blood pressure at home predicts erythropoietin-induced
hypertension in patients with chronic renal diseases.. Clin Exp Nephrol.

[18] Kuriyama S, Otsuka Y, Iida R, Matsumoto K, Tokudome G, Hosoya T (2005). Morning blood pressure predicts hypertensive organ damage in
patients with renal diseases: effect of intensive antihypertensive therapy
in patients with diabetic nephropathy.. Intern Med.

[19] Uchida H, Nakamura Y, Kaihara M, Norii H, Hanayama Y, Makino H (2008). The MUSCAT study: a multicenter PROBE study comparing the effects
of angiotensin II type-1 receptor blockers on self-monitored home blood
pressure in patients with morning hypertension: study design and background
characteristics.. Hypertens Res.

[20] Martin LC (2014). Botucatu.

[21] Sociedade Brasileira de Cardiologia, Sociedade Brasileira de
Hipertensão, Sociedade Brasileira de Nefrologia. (2011). V diretrizes de monitoração ambulatorial da pressão arterial
(MAPA) e III diretrizes de monitoração residencial da pressão arterial
(MRPA) [V Guidelines for ambulatory blood pressure monitoring (ABPM) and III
Guidelines for home blood pressure monitoring (HBPM)].. Arq Bras Cardiol..

[22] STROBE (2023). Statement-checklist of items that should be included in reports of
observational studies [Internet].

[23] Nobre F, Mion D, Gomes MAM, Barbosa ECD, Rodrigues CIS, Neves MFT (2018). 6^a^ Diretrizes de Monitorização Ambulatorial da Pressão
Arterial e 4^a^ Diretrizes de Monitorização Residencial da Pressão
Arterial.. Arq Bras Cardiol..

[24] Kidney Disease: Improving Global Outcomes (KDIGO) CKD Work
Group. (2013). KDIGO 2012 clinical practice guideline for the evaluation and
management of chronic kidney disease.. Kidney Int Suppl..

[25] Agarwal R, Andersen MJ (2005). Correlates of systolic hypertension in patients with chronic
kidney disease.. Hypertension.

[26] Portaluppi F, Montanari L, Massari M, Di Chiara V, Capanna M (1991). Loss of nocturnal decline of blood pressure in hypertension due
to chronic renal failure.. Am J Hypertens..

[27] Baumgart P, Walger P, Gemen S, von Eiff M, Raidt H, Rahn KH (1991). Blood pressure elevation during the night in chronic renal
failure, hemodialysis and after renal transplantation.. Nephron J.

[28] Velasquez MT, Beddhu S, Nobakht E, Rahman M, Raj DS (2016). Ambulatory blood pressure in chronic kidney disease: ready for
prime time?. Kidney Int Rep.

[29] Timio M, Lolli S, Verdura C, Monarca C, Merante F, Guerrini E (1993). Circadian blood pressure changes in patients with chronic renal
insufficiency: a prospective study.. Ren Fail.

[30] Tang H, Gong WY, Zhang QZ, Zhang J, Ye ZC, Peng H (2016). Prevalence, determinants, and clinical significance of masked
hypertension and white-coat hypertension in patients with chronic kidney
disease.. Nephrology.

