# Supplementary material for: Association between chronic kidney disease stages and changes in ambulatory blood pressure monitoring parameters
Source: J Bras Nefrol. 2024 Jun 17;46(3):e20230066. doi: 10.1590/2175-8239-JBN-2023-0066en (PMC11205976; doi:10.1590/2175-8239-JBN-2023-0066en)
Supplement: Translation

# Translation: Associação entre estágios da doença renal crônica e alterações dos parâmetros da monitorização ambulatorial da pressão arterial

**DOI:** 10.1590/2175-8239-JBN-2023-0066pt

**Authors:** André Murad Nagahama, Vanessa dos Santos Silva, Vanessa Burgugi Banin, Roberto Jorge da Silva Franco, Pasqual Barretti, Silmeia Garcia Zanati Bazan, Luis Cuadrado Martin

**Affiliations:** 1 Universidade Estadual Paulista “Júlio de Mesquita Filho”, Faculdade de Medicina, Botucatu, SP, Brasil.

**Keywords:** Doença Renal Crônica, Insuficiência Renal Crônica, Monitorização Ambulatorial da Pressão Arterial, Monitoramento Ambulatorial da Pressão Arterial, Hipertensão, Hipertensão Arterial, Ritmo Circadiano

## Abstract

**Introdução:**

A avaliação da pressão arterial (PA) tem impacto no manejo da hipertensão arterial (HA) na doença renal crônica (DRC). O portador de DRC apresenta padrão específico de comportamento da PA ao longo da monitorização ambulatorial da pressão arterial (MAPA).

**Objetivos:**

O objetivo do corrente estudo é avaliar as associações entre os estágios progressivos da DRC e alterações da MAPA.

**Metodologia:**

Trata-se de um estudo transversal com 851 pacientes atendidos nos ambulatórios de um hospital universitário que foram submetidos ao exame de MAPA no período de janeiro de 2004 a fevereiro de 2012 para avaliar a presença e o controle da HA. Os desfechos considerados foram os parâmetros de MAPA. A variável de interesse foi o estadiamento da DRC. Foram considerados como fatores de confusão idade, sexo, índice de massa corporal, tabagismo, causa da DRC e uso de anti-hipertensivos.

**Resultados:**

A PA sistólica (PAS) se associou aos estágios 3b e 5 da DRC, independentemente das variáveis de confusão. Pressão de pulso se associou apenas ao estágio 5. O coeficiente de variação da PAS se associou progressivamente aos estágios 3a, 4 e 5, enquanto o coeficiente de variação da pressão arterial diastólica (PAD) não demonstrou associação. O descenso da PAS obteve associação com estágios 2, 4 e 5, e o descenso da PAD, com os 4 e 5. Demais parâmetros da MAPA não obtiveram associação com os estágios da DRC após os ajustes.

**Conclusão:**

Estágios mais avançados da DRC associaram-se a menor descenso noturno e a maior variabilidade da pressão arterial.

## Introdução

A prevalência da hipertensão arterial (HA) é elevada^1^, e essa condição clínica compromete coração, vasos sanguíneos, cérebro e rins^2^. A prevalência da doença renal crônica (DRC) no Brasil é estimada em cerca de 10% da população^2^, e destes, aproximadamente 150 mil estão no estágio progressivo mais avançado, necessitando de diálise^3^.

A HA é altamente prevalente na DRC^4^. O elo fisiopatológico entre HA e DRC é conhecido, sendo a HA o maior fator de risco para DRC no Brasil^3^, já que a hipertensão e a injúria renal estabelecem um ciclo de causa e consequência que piora o prognóstico^5^. Dentre as características fisiopatogênicas da HA na DRC, podemos citar a retenção de sódio, hiperatividade simpática e do sistema renina-angiotensina-aldosterona^6^. Essas particularidades se associam a características do comportamento da pressão arterial (PA) e seu padrão circadiano^7^.

Assim, o controle da PA é crucial, já que diminui danos estruturais e funcionais dos néfrons e melhora a expectativa de vida do paciente com doença renal^6^, o que enfatiza a avaliação precisa da PA na DRC. A definição do tipo de HA em normotensão, hipertensão verdadeira, hipertensão do avental branco e hipertensão mascarada é essencial para o manejo dos pacientes^8^.

A monitorização ambulatorial da pressão arterial (MAPA) é a melhor forma disponível na clínica para avaliar a PA do paciente ao longo de 24 horas^8,9,10,11,12^. O portador de DRC apresenta padrão específico de comportamento da PA ao longo de 24 horas, a saber: diminuição do descenso noturno ou até aumento da PA durante o sono.

Alguns estudos avaliaram esses padrões, no entanto ainda são escassos. Uma metanálise^13^ identificou apenas 6 estudos^14,15,16,17,18,19^ que avaliaram a prevalência de hipertensão do avental branco e hipertensão mascarada em doentes renais crônicos. Essa metanálise identificou altas prevalências de hipertensão mascarada e hipertensão do avental branco em pacientes com DRC, porém, dentre os estudos analisados, o mais significativo deles havia incluído apenas 290 pacientes^15^. É importante salientar que esses trabalhos não levaram em conta variáveis de confusão.

Não identificamos trabalhos sobre alterações específicas da MAPA em DRC que tenham avaliado separadamente cada um dos estágios específicos dos progressivos estágios da doença, nem trabalhos que compararam com grupos controle sem DRC ou fizeram ajustes estatísticos para as variáveis de confusão.

Portanto, o objetivo do corrente estudo é avaliar as associações entre estágios progressivos da DRC e alterações da MAPA considerando variáveis de confusão.

## Métodos

Analisamos, neste trabalho transversal, 851 pacientes dos ambulatórios de cardiologia, endocrinologia, clínica médica e nefrologia do Hospital das Clínicas da Faculdade de Medicina de Botucatu. Esses pacientes foram submetidos ao exame de MAPA no período de janeiro de 2004 a fevereiro de 2012, indicado para avaliação diagnóstica e controle da HA^20^. A maioria dos pacientes realizava tratamento medicamentoso e isso foi considerado nas análises estatísticas.

Os desfechos considerados foram os seguintes parâmetros de MAPA: médias de pressão arterial sistólica (PAS), pressão arterial diastólica (PAD), pressão de pulso (PP); porcentagem de descenso ao sono da PAS e PAD; variabilidade da PA; fenótipo da HA (normotensão, hipertensão sustentada, hipertensão do avental branco e hipertensão mascarada). A variável de interesse é o estadiamento da DRC. Consideramos fatores de confusão: idade, sexo, índice de massa corporal (IMC), tabagismo e causa da DRC.

Foram considerados critérios de inclusão pacientes maiores de 18 anos que realizaram MAPA no período de estudo. Os critérios de exclusão foram: pacientes que tenham realizado MAPAs tecnicamente inadequados segundo as V Diretrizes brasileiras de monitorização ambulatorial da pressão arterial e Diretrizes de monitorização residencial da pressão arterial; grávidas; portadores de transplante renal; parkinsonianos; portadores de fibrilação atrial; dados de MAPA incompletos; repetições do exame; e ausência de dados para estabelecer o estágio da DRC.

Foram anotadas a PAS e a PAD de consultório obtidas imediatamente antes da realização do exame de MAPA. Os registros de creatinina e exames de urina feitos próximos ao período da realização da MAPA (no máximo 3 meses antes ou depois da MAPA) foram anotados. A taxa de filtração glomerular (TFG) foi calculada através da fórmula “Chronic Kidney Disease Epidemiology Collaboration” (CKD-EPI).

Os exames da MAPA foram realizados com o Spacelabs^®^ 90202. Padronizou-se a MAPA a partir das V Diretrizes brasileiras de monitoração ambulatorial da pressão arterial (MAPA) e III Diretrizes de monitoração residencial da pressão arterial^21^. Registrou-se via software as médias da PAS e PAD em todo o período de exame, no período de vigília e de sono. Foram calculados os descensos da PAS e da PAD durante o sono. A variabilidade da pressão arterial foi quantificada pelo desvio-padrão das pressões, bem como pelo coeficiente de variação.

Este trabalho está de acordo com o *Strobe Statement* ^22^, bem como com a Resolução 466/12 do Conselho Nacional de Saúde, que é compatível com a declaração de Helsinque. Também foi aprovado pelo comitê de ética em pesquisa local e foi obtida dispensa do termo de consentimento informado.

## Definições

Utilizamos como definição para hipertensão do avental branco: pressão arterial no consultório maior ou igual a 140/90 mmHg e medições da MAPA no período de vigília menor ou igual a 135/85 mmHg. Diante das novas considerações das VI Diretrizes brasileiras de monitorização ambulatorial da pressão arterial (MAPA)^23^, considerou-se também a PA durante o sono. Dessa forma, incluiu-se na definição a média da PA de 24 horas maior ou igual a 130/80 mmHg.

A definição de hipertensão mascarada utilizada foi: PA no consultório menor que 140/90 mmHg e medições da MAPA no período de vigília maior ou igual a 135/85 mmHg. Diante das novas considerações das VI Diretrizes brasileiras de monitorização ambulatorial da pressão arterial^23^, considerou-se também a PA durante o sono. Dessa forma, incluiu-se na definição a PA no período de sono maior ou igual a 120/70 mmHg.

A hipertensão sustentada foi definida quando a PA no consultório foi maior ou igual a 140/90 mmHg e as medições da MAPA no período de vigília foram maiores ou iguais a 135/85 mmHg; enquanto a normotensão quando a PA no consultório foi menor que 140/90 mmHg e as medições da MAPA no período de vigília foram menores que 135/85 mmHg. Os dados também foram reanalisados pelas VI Diretrizes brasileiras de monitorização ambulatorial da pressão arterial^23^.

A DRC foi definida pelo KDIGO 2012^24^ como filtração glomerular menor que 60 mL/min/1,73m^2^, ou presença de proteinúria por mais de 3 meses. Além disso, a DRC foi classificada levando em consideração a sua etiologia e a TFG.

Os diferentes estágios de TFG na CKD são: G1 definido por uma TFG ≥ 90 mL/min/1,73m^2^. G2, TFG entre 60 e 89 mL/min/1,73m^2^. G3a, TFG entre 45 e 59 mL/min/1,73m^2^. G3b é caracterizado por uma TFG entre 30 e 44 mL/min/1,73m^2^. G4, TFG entre 15 e 29 mL/min/1,73m^2^. Por fim, G5 é definido por uma TFG menor que 15 mL/min/1,73m^2^.

## Análise Estatística

Os dados categóricos foram expressados em números absolutos e frequências. As variáveis não categóricas de distribuição normal foram expressas em média ± desvio-padrão. A normalidade dos dados foi verificada pelo teste de Kolmogorov-Smirnov. As variáveis contínuas de distribuição normal foram comparadas por ANOVA de uma via. As variáveis contínuas de distribuição não gaussianas foram submetidas ao teste de Kruskal-Wallis. As variáveis categóricas foram comparadas pelo teste de χ^2^. Aplicamos o modelo múltiplo de regressão linear generalizada à variável de interesse, bem como aos seguintes fatores de confusão: idade, sexo, IMC, tabagismo, causa da DRC e quantidade de classes anti-hipertensivas. Os dados foram discutidos considerando o nível de p < 0,05.

## Resultados

Foram realizados 1.308 exames de MAPA ao longo do período do estudo. Destes, 52 foram excluídos porque não preencheram as condições técnicas de validação do procedimento. Além disso, 92 pacientes eram menores de 18 anos, 18 eram pacientes com rins transplantados e 149 foram exames repetidos, os quais também foram excluídos. Por fim, foram excluídos mais 146 pacientes que não apresentavam todos os dados de creatinina ou resultados de exames de urina. Dessa forma, foram incluídos nas análises 851 pacientes (Figura 1).

**Figura 1.** Fluxograma de inclusão de pacientes.

A idade foi de 55 ± 16,2 anos; 42,4% eram do sexo masculino; 6,6% eram afrodescendentes; asiáticos e brancos compuseram 0,9% e 92,5%, respectivamente. Dos 682 pacientes, 74 (10,9%) eram tabagistas ativos, enquanto 156 (22,9%) dos 682 eram ex-tabagistas.

Da amostra total, 475 pacientes apresentavam DRC. Dentre estes 475 com DRC, 168 (35,6%) tinham como causa o diabetes; 227 (47,8%), hipertensão arterial; 32 (6,7%) apresentavam causas glomerulares; e 47 (9,9%), outras causas.

Quanto ao estadiamento da DRC, 376 dos 851 pacientes não tinham DRC. Dentre os 475 que tinham, 45 eram do estágio G1; 54, do estágio G2; 97, do estágio G3a; 89, do estágio G3b; 71, do estágio G4; e 119, do estágio G5 (Tabela 1).

**Tabela 1.** Dados clínicos e demográficos de 851 pacientes submetidos à monitorização ambulatorial da pressão arterial

	Não DRC (n = 376)	Estágios da DRC						P
		1 (n = 45)	2 (n = 54)	3a (n = 97)	3b (n = 89)	4 (n = 71)	5 (n = 119)	
Idade (anos)	49 ± 15,0	40 ± 13,8	54 ± 13,7	61 ± 13,6	66 ± 14,7	65 ± 11,6	61 ± 14,2	<0,001
Sexo masculino (%)	40	24	38	45	49	49	54	0,010
Afrodescendentes (%)	7	11	0	4	4	7	10	0,154
IMC (Kg/m^2^)	29 ± 5,3	27 ± 5,9	29 ± 6,8	28 ± 6,0	28 ± 6,6	28 ± 6,9	25 ± 3,7	0,344
Tabagistas ativos *(%)	34/293 (11)	5/33 (15)	1/42 (2)	3/74 (4)	6/73 (8)	11/60 (18)	14/107 (13)	0,062
Ex-tabagistas *(%)	52/293 (17)	7/33 (21)	12/42 (28)	23/74 (31)	24/73 (32)	13/60 (21)	24/107 (22)	0,056
Diabéticos (%)	21	20	46	38	31	42	49	<0,001
Dislipidemia *(%)	102/281 (36)	11/31(35)	16/45 (35)	46/78 (58)	49/77 (63)	43/63 (68)	45/91 (49)	<0,001

Abreviações – DRC: doença renal crônica; IMC: índice de massa corpórea;

* (%) = casos positivos/casos totais com o dado disponível (%).

O estadiamento da doença renal crônica se associou a idade (p < 0,001), sexo masculino (p = 0,01), diabetes (p < 0,001) e dislipidemia (p < 0,001). Etnia, IMC (p = 0,344) e tabagismo (p = 0,059) não tiveram associação estatisticamente significante com a evolução da DRC (Tabela 1), porém foram incluídos no modelo múltiplo de análise. A mediana e o intervalo interquartílico do número de classes de anti-hipertensivos empregadas em cada estágio estão ilustrados na Figura 2.

**Figura 2.** Diagrama de caixa da distribuição de anti-hipertensivos nos diferentes estágios da doença renal crônica.

Por meio da MAPA, foi possível observar a seguinte distribuição de fenótipos de hipertensão: 230 eram normotensos; 140 tinham hipertensão mascarada; 134 tinham hipertensão do avental branco; 347 eram hipertensos verdadeiros (Tabela 2).

**Tabela 2.** Resultados de pressão arterial de 851 pacientes submetidos à monitorização ambulatorial da pressão arterial

	Não DRC (n = 376)	Estágios da DRC						P
		1 (n = 45)	2 (n = 54)	3a (n = 97)	3b (n = 89)	4 (n = 71)	5 (n = 119)	
PAS cons. (mmHg)	137 ± 22,1	137 ± 24,8	137 ± 21,9	141 ± 24,5	141 ± 25,6	143 ± 27,4	148 ± 28,9	0,007
PAD cons. (mmHg)	89 ± 14,3	87 ± 13,3	86 ± 13,4	87 ± 16,4	84 ± 17,7	84 ± 15,9	87 ± 16,1	0,156
PAS 24h (mmHg)	129 ± 14,6	127 ± 15,7	131 ± 18,0	129 ± 15,9	128 ± 16,6	134 ± 22,3	141 ± 23,3	<0,001
CV PAS 24h (%)	10,1 ± 2,40	9,7 ± 2,38	10,3 ± 2,74	11,1 ± 3,03	10,9 ± 3,19	11,8 ± 3,60	11,9 ± 3,24	<0,001
PAD 24h (mmHg)	79 ± 11,1	77 ± 10,3	78 ± 10,6	76 ± 9,9	73 ± 13,0	76 ± 13,0	79 ± 14,7	0,001
CV PAD 24h (%)	13,9 ± 3,21	14,5 ± 3,38	13,6 ± 3,12	14,3 ± 3,57	14,0 ± 3,26	13,9 ± 3,53	13,7 ± 3,74	0,723
PP 24h (mmHg)	50 ± 10,3	49 ± 11,1	52 ± 13,8	52 ± 12,1	54 ± 13,2	58 ± 15,8	61 ± 16,6	<0,001
PAS vig. (mmHg)	132 ± 14,9	130 ± 15,8	133 ± 17,6	131 ± 16,0	130 ± 16,6	135 ± 21,7	141 ± 23,0	<0,001
PAD vig. (mmHg)	82 ± 11,5	81 ± 11,0	81 ± 11,4	79 ± 10,6	76 ± 13,4	78 ± 13,2	80 ± 14,8	0,001
PAS sono (mmHg)	121 ± 15,8	119 ± 16,5	126 ± 20,4	123 ± 18,7	122 ± 18,0	131 ± 27,0	139 ± 26,1	<0,001
PAD sono (mmHg)	71 ± 11,8	69 ± 10,0	73 ± 11,0	70 ± 11,1	68 ± 13,7	71 ± 14,9	77 ± 16,1	<0,001
Desc. PAS (%)	8 ± 7,1	8 ± 7,5	5 ± 7,6	5 ± 10,1	5 ± 6,0	3 ± 10,8	1 ± 7,5	<0,001
Desc. PAD (%)	13 ± 8,2	15 ± 7,6	9 ± 9,9	10 ± 9,9	10 ± 7,7	7 ± 12,1	4 ± 7,8	<0,001
DP PAS (mmHg)	13 ± 3,4	12 ± 3,3	13 ± 4,6	14 ± 3,9	13 ± 4,4	15 ± 5,3	16 ± 4,7	<0,001
DP PAD (mmHg)	10 ± 2,3	11 ± 2,6	10 ± 2,3	10 ± 2,6	10 ± 2,5	10 ± 2,7	10 ± 2,3	0,239
Fenótipos								
Normotensos (%)	81(21)	9(20)	10(19)	21(22)	23(26)	15(21)	13(11)	0,186
HM (%)	93(25)	13(29)	16(30)	14(14)	17(19)	20(28)	25(21)	0,180
HAB (%)	41(11)	6(13)	4(7)	24(25)	12(13)	2(3)	7(6)	<0,001
Hipertensos (%)	161(43)	17(38)	24(44)	38(39)	37(42)	34(48)	74(62)	0,006
Acurácia da pressão de consultório (%)	64	58	63	61	67	69	73	0,415

Abreviações – DRC: doença renal crônica; PAS: pressão arterial sistólica; PAD: pressão arterial diastólica; cons.: consultório; CV: coeficiente de variação; PP: pressão e pulso; vig. vigília; desc.: descenso; DP: desvio-padrão; HM: hipertensão mascarada; HAB: hipertensão do avental branco. Fenótipo tradicional: 5^a^ Diretriz de MAPA; fenótipo atual: 6^a^ Diretriz de MAPA.

Em relação às diferenças de PA nos progressivos estágios da DRC (Tabela 2), ocorreu elevação progressiva da PAS de consultório à medida que a lesão renal progredia (p = 0,007). Todavia, a PAD de consultório não se associou com a evolução da DRC (p = 0,156). Quanto aos parâmetros da MAPA, com exceção do coeficiente de variação da PAD de 24 horas e desvio-padrão da PAD, pudemos observar uma associação entre diferentes estágios evolutivos da DRC e piora dos parâmetros do exame de MAPA. Houve associação entre os fenótipos de hipertensão e o estágio da DRC para hipertensão do avental branco e hipertensão sustentada. A acurácia da pressão de consultório não se associou aos estágios mais avançados da DRC (Tabela 2).

A partir da realização das regressões lineares generalizadas, obtivemos uma série de associações entre o estágio da DRC e os resultados do exame da MAPA ajustadas para as variáveis de confusão: sexo, tabagismo, dislipidemia, diabetes, idade e quantidade de classes de anti-hipertensivos. A pressão arterial sistólica à MAPA se associou aos estágios 3a e 5 da DRC, independentemente das variáveis de confusão incluídas no modelo (Tabela 3). Observou-se uma diminuição do valor da PAS de 24 horas para o estágio 3b, enquanto que para o estágio 5 observou-se aumento do seu valor. A diminuição da pressão arterial diastólica à MAPA se associou apenas ao estágio 3b. O aumento da PP de 24 horas da MAPA apresentou associação estatisticamente significante ao estágio 5 da DRC, independentemente das variáveis de ajuste (Tabela 3).

**Tabela 3.** Modelo de regressão linear geral tendo como variável desfecho a pressão arterial sistólica, diastólica e pressão de pulso de 24 horas de 851 pacientes submetidos à monitorização ambulatorial da pressão arterial ajustado para idade, sexo, tabagismo, diabetes, dislipidemia e quantidade de classes de anti-hipertensivos

	β	95% Intervalo de Confiança de Wald		p
		Inferior	Superior	
PAS				
Estágio 5	5,421	0,958	9,885	0,017
Estágio 4	–0,362	–5,646	4,921	0,893
Estágio 3b	–6,043	–11,028	–1,058	0,018
Estágio 3a	–2,829	–7,605	1,947	0,246
Estágio 2	–0,690	–6,667	5,288	0,821
Estágio 1	0,332	–7,568	6,923	0,929
Sem DRC	Referência			
PAD				
Estágio 5	0,578	–2,449	3,605	0,708
Estágio 4	–2,469	–6,042	1,124	0,179
Estágio 3b	–3,751	–7,132	–0,371	0,030
Estágio 3a	–1,135	–4,373	2,104	0,492
Estágio 2	–0,625	–4,679	3,428	0,762
Estágio 1	–3,365	–8,285	1,554	0,180
Sem DRC	Referência			
PP				
Estágio 5	4,843	1,802	7,884	0,002
Estágio 4	2,096	–1,506	5,696	0,254
Estágio 3b	–2,292	–5,688	1,104	0,186
Estágio 3a	–1,694	–4,948	1,559	0,307
Estágio 2	–0,064	–4,136	4,008	0,975
Estágio 1	3,034	–1,908	7,975	0,229
Estágio 0	Referência			

Sobre o coeficiente de variação da PAS de 24 horas, o aumento deste se associou aos estágios 3a, 4 e 5 da DRC nas regressões, mesmo considerando as outras variáveis de confusão. O coeficiente de variação da PAD de 24 horas da MAPA, entretanto, não se associou a nenhum estágio da DRC (Tabela 4).

**Tabela 4.** Modelo de regressão linear geral tendo como variável desfecho o coeficiente de variação da pressão arterial sistólica e diastólica e descenso da pressão arterial sistólica e diastólica de 851 pacientes submetidos à monitorização ambulatorial da pressão arterial ajustado para idade, sexo, tabagismo, diabetes e dislipidemia e quantidade de classes de anti-hipertensivos

	β	95% Intervalo de Confiança de Wald		p
		Inferior	Superior	
CV PAS				
Estágio 5	2,032	1,282	2,782	<0,001
Estágio 4	1,138	0,250	2,026	0,001
Estágio 3b	0,155	–0,683	0,993	0,716
Estágio 3a	0,977	0,175	1,780	0,017
Estágio 2	0,213	–0,791	1,218	0,677
Estágio 1	–0,171	–1,390	1,049	0,784
Sem DRC	Referência			
CV PAD				
Estágio 5	0,778	–0,137	1,693	0,095
Estágio 4	0,484	–0,598	1,567	0,380
Estágio 3b	0,437	–0,584	1,459	0,401
Estágio 3a	0,655	–0,324	1,634	0,190
Estágio 2	–0,232	–1,457	0,993	0,711
Estágio 1	0,138	–1,348	1,625	0,855
Sem DRC	Referência			
Descenso PAS				
Estágio 5	–4,272	–6,306	–2,238	<0,001
Estágio 4	–4,494	–6,901	–2,087	<0,001
Estágio 3b	–1,323	–3,595	0,948	0,253
Estágio 3a	0,253	–1,923	2,429	0,820
Estágio 2	–2,997	–5,720	–0,274	0,031
Estágio 1	–1,152	–4,457	2,153	0,494
Sem DRC	Referência			
Descenso PAD				
Estágio 5	–5,370	–7,694	–3,046	<0,001
Estágio 4	–3,576	–6,327	–0,825	0,011
Estágio 3b	0,030	–2,566	2,629	0,982
Estágio 3a	5,474	–1,940	3,034	0,667
Estágio 2	–2,436	–5,548	0,677	0,125
Estágio 1	–0,386	–4,164	3,391	0,841
Sem DRC	Referência			

Por fim, sobre os descensos da PA ao longo do exame: a diminuição do descenso da PAS se associou aos estágios 2, 4 e 5 de forma independente. No entanto, a redução do valor do descenso da PAD se associou somente aos dois estágios mais avançados da DRC (Tabela 4).

## Discussão

Este estudo verificou a associação entre estágios da DRC e anormalidades observadas na MAPA. Foi possível observar que estágios mais avançados da DRC estavam associados progressivamente a menor descenso noturno e maior variabilidade da pressão arterial. PAS de 24 horas e PP de 24 horas estavam associadas a nível mais avançado da DRC (estágio V). O diferencial atributo do corrente estudo é que todas essas associações foram observadas independentemente de sexo, idade, tabagismo, diabetes e, inclusive, quantidade de classes anti-hipertensivos.

Um estudo transversal, abarcando 10.271 pacientes hipertensos, observou que a pressão de pulso de pacientes renais crônicos era significantemente maior comparada à PP dos hipertensos sem doença renal^7^. Nesse estudo, a maior diferença entre os dois grupos pesquisados foi a prevalência do padrão “riser” (17,6% em pacientes com CKD × 7,1% em pacientes não CKD), isto é, no qual a média da pressão sistólica do sono é maior do que a média da pressão sistólica em vigília. Esses dados são compatíveis com os dados do corrente trabalho, entretanto esse estudo transversal não realizou os ajustes para variáveis de confusão, como sexo, idade, tabagismo e presença de diabetes.

Agarwal & Andersen^25^, em um estudo com 232 veteranos, considerando 17 variáveis de confusão, observaram que a PAS não se associou com o declínio da filtração glomerular quando foram realizados os ajustes cabíveis. Entretanto, os autores observaram forte associação entre a PAS de 24 horas da MAPA e a proteinúria. Além disso, o padrão dipper, de maneira geral, diminuía à medida que o grau de DRC aumentava, porém não linearmente. Em nosso estudo, o padrão não dipper se associou à queda da taxa de filtração glomerular, isto é, aos estágios mais avançados da DRC (estágios 2, 4 e 5), como esperado diante de evidências da literatura^26,27^. Em nosso trabalho, os pacientes dos estágios 3b e 5 apresentaram associação com menor e maior PAS, respectivamente. Interessante notar que o trabalho de Agarwal & Andersen não incluiu pacientes dialíticos (estágio 5)^25^.

A variabilidade da PA em pacientes renais crônicos é aumentada, cuja explicação acredita-se acontecer por conta de um mau funcionamento dos barorreceptores e descontrole do balanço entre atividade simpática e parassimpática^28^. Timio et al.^29^, em um estudo prospectivo de 1993, observaram que a variabilidade da pressão arterial em doentes renais era superior à dos controles. Em nosso trabalho, ao realizar regressão linear generalizada, o coeficiente de variação da PAS se associou apenas com os estágios mais avançados da DRC (estágios 3a, 4 e 5), enquanto que para a PAD não se observou nenhuma associação em nenhum estágio da doença estudada.

No presente estudo, paradoxalmente, a acurácia da pressão aferida em consultório não se associou aos estadiamentos mais graves de DRC, mesmo considerando a classificação antiga e a nova para hipertensão mascarada e hipertensão do avental branco, que abordam diferentemente a utilização da pressão noturna e diurna da MAPA. Acreditamos que essa diferença com a literatura^30^ seja por causa da realização de uma medida de PA padronizada no nosso estudo, diminuindo possíveis interferências externas, como consumo de cafeína, fumo, exercício físico e negligência quanto ao tempo de repouso antes da aferição.

Devemos reconhecer algumas limitações neste estudo. Os indivíduos estudados eram todos provenientes da região de Botucatu, no interior do estado de São Paulo. Dessa forma, obtivemos uma amostra demográfica que não é concordante com os parâmetros demográficos do Brasil como um todo. Acreditamos que isso se deva às características da cidade em questão. Também não possuíamos nenhuma forma de avaliação sobre adesão ao tratamento da HA e DRC apesar de sabermos o esquema terapêutico de cada paciente. Outro ponto limitante deste trabalho foi, apesar de termos o exame de urina qualitativa avaliada pela Urina I, não termos obtido os dados relacionados à proteinúria quantitativa de uma porção considerável dos pacientes, o que impediu a classificação completa do KDIGO 2012. Por fim, a prevalência de hipertensão mascarada pode ter sido subestimada, tendo em vista que neste trabalho não foi realizado sistematicamente exame de MAPA em todos os renais crônicos que apresentaram pressão arterial de consultório normal, apenas nos renais crônicos que fizeram MAPA em nossa instituição. Por outro lado, o mesmo viés poderia ocorrer entre os pacientes que não apresentaram DRC em nossa casuística, o que poderia equilibrar os vieses.

Também podemos apontar aspectos positivos no corrente trabalho que ainda não foram explorados na literatura. Primeiramente, tratando-se de um estudo transversal, conseguimos abarcar uma amostra extensa de 851 pacientes com uma boa representatividade ao longo dos diferentes estágios da DRC. Na literatura, encontramos raros estudos que analisassem de maneira tão completa os parâmetros de MAPA concomitantemente aos diferentes estágios da DRC. Além disso, poucos estudos consideraram variáveis de confusão na análise de seus dados.

## Conclusão

Em nossa casuística, estágios mais avançados da DRC associaram-se a menor descenso noturno e a maior variabilidade da pressão arterial. Elevações da PAS e da PP foram verificadas apenas no estágio 5. Por outro lado, a diminuição da PAS de 24 horas se associou a um estágio intermediário da DRC (estágio 3b), mesmo levando em conta fatores de confusão. Paradoxalmente, a acurácia da pressão aferida em consultório não se associou aos estágios mais avançados da DRC.

Nesse contexto, o corrente trabalho abrangeu um número maior de pacientes e dados a serem analisados, como também avaliou de forma detalhada as relações entre os diferentes estágios da DRC e as principais características específicas da MAPA, levando em conta eventuais fatores de confusão.

Desta forma, pudemos explorar adequadamente como os diferentes estágios da DRC se associam aos parâmetros do exame de MAPA.
